# Ebstein's Anomaly: "The One and a Half Ventricle Heart"

**DOI:** 10.21470/1678-9741-2018-0100

**Published:** 2018

**Authors:** Amber Malhotra, Vishal Agrawal, Kartik Patel, Mausam Shah, Kamal Sharma, Pranav Sharma, Sumbul Siddiqui, Nilesh Oswal, Himani Pandya

**Affiliations:** 1 Department of Cardiovascular and Thoracic Surgery, U. N. Mehta Institute of Cardiology and Research Centre (affiliated to BJ medical college, Ahmedabad), Gujarat, India.; 2 Department of Cardiology, U. N. Mehta Institute of Cardiology and Research Centre (affiliated to BJ medical college, Ahmedabad), Gujarat, India.; 3 Department of Research, U. N. Mehta Institute of Cardiology and Research Centre (affiliated to BJ medical college, Ahmedabad), Gujarat, India.

**Keywords:** Ebstein Anomaly, Heart Defects, Congenital, Cardiovascular Surgical Procedures

## Abstract

**Objective:**

Ebstein's anomaly remains a relatively ignored disease. Lying in the 'No Man's land' between congenital and valve surgeons, it largely remains inadequately studied. We report our short-term results of treating it as a 'one and a half ventricle heart' and propose that the true tricuspid annulus (TTA) 'Z' score be used as an objective criterion for estimation of 'functional' right ventricle (RV).

**Methods:**

22 consecutive patients undergoing surgery for Ebstein's anomaly were studied. Echocardiography was performed to assess the type and severity of the disease, tricuspid annular dimension and its 'Z' score. Patients were operated by a modification of the cone repair, with addition of annuloplasty, bidirectional cavopulmonary shunt (BCPS) and right reduction atrioplasty to provide a comprehensive repair. TTA 'Z' score was correlated later with postplication indexed residual RV volume.

**Results:**

There was one (4.5%) early and no late postoperative death. There was a significant reduction in tricuspid regurgitation grading (3.40±0.65 to 1.22±0.42, *P*<0.001). Residual RV volume reduced to 71.96±3.8% of the expected volume and there was a significant negative correlation (rho −0.83) between TTA 'Z' score and indexed residual RV volume. During the follow-up of 20.54±7.62 months, the functional class improved from 2.59±0.7 to 1.34±0.52 (*P*<0.001).

**Conclusion:**

In Ebstein's anomaly, a higher TTA 'Z' score correlates with a lower postplication indexed residual RV volume. Hence, a complete trileaflet repair with offloading of RV by BCPS (when the TTA 'Z' score is >2) is recommended. The short-term outcomes of our technique are promising.

**Table:** 

Abbreviations, acronyms & symbols	
ACE	= Angiotensin-converting-enzyme
ATL	= Anterior tricuspid leaflet
BCPS	= Bidirectional cavopulmonary shunt
CPB	= Cardiopulmonary bypass
ICU	= Intensive care unit
LVEF	= Left ventricular ejection fraction
NYHA	= New York Heart Association
PTL	= Posterior tricuspid leaflet
RAA	= Right atrial appendage
RV	= Right ventricle
STL	= Septal tricuspid leaflet
TAPSE	= Tricuspid annular plane systolic excursion
TR	= Tricuspid regurgitation
TTA	= True tricuspid annulus

## INTRODUCTION

In 1866, Wilhelm Ebstein described a complex congenital cardiac anomaly during the autopsy of a 19-year-old cyanotic man^[1]^. The complex lesion was named Ebstein's anomaly and included septal and posterior leaflet adherence to the underlying myocardium with downward displacement of the functional tricuspid annulus, resulting in dilatation of atrialized portion of right ventricle (RV) and true tricuspid annulus (TTA; the right atrioventricular junction)^[2]^. It is a rare congenital cardiac anomaly, occurring in approximately 1 per 200,000 live births and accounting for <1% of all cases of congenital heart disease^[2,3]^. The complexity of the anomaly led to several classifications being proposed, especially those proposed by Carpentier et al.^[4]^ and Celermajer et al.^[5]^. Various surgical techniques have been described for the repair of this complex pathology, depending on the surgeon's understanding of anatomical and functional alterations that involve the tricuspid valve, right atrium, RV and conduction system^[6-11]^. Most of the techniques involved repair in the leaflet and annular level with plication of the atrialized RV either horizontally or vertically, resulting in a monocuspid or bicuspid valve^[7,8,12]^. We describe a standardized technique of a physiologically and anatomically complete trileaflet repair of Ebstein's anomaly. In view of the inevitable association of RV dysfunction with tricuspid deformity, a comprehensive repair of all subcomponents of the anomaly has been practised at our institute for a possible long-term event-free survival of these patients. It includes: 1) Plication of the atrialized right ventricle; 2) Reduction of tricuspid valve annulus resulting in neoannulus; 3) Trileaflet repair of tricuspid valve; 4) Tricuspid ring annuloplasty; 5) Right atrial reduction ± MAZE; 6) Bidirectional cavopulmonary shunt (BCPS). To prove the need for BCPS shunt, we measured the residual volume of RV after plication and correlated it with the expected indexed RV volume.

## METHODS

Between January 2012 to July 2016, 22 consecutive patients underwent surgery for Ebstein's anomaly at the Department of Cardiothoracic Surgery, U.N. Mehta Institute of Cardiology & Research Centre, Ahmedabad.

### Preoperative Evaluation

Preoperative data including age, sex, previous cardiac surgery, cyanosis, palpitations, dyspnea on exertion, pedal edema, hepatomegaly, presence of other associated cardiac anomalies, arrhythmias, renal dysfunction, congestive cardiac failure, need for ventilatory support, etc., were collected. Preoperative echocardiography using Vivid i (GE Healthcare) was performed to evaluate the severity and type of disease, degree of tricuspid regurgitation (TR), true tricuspid annular dimension with 'Z' score, tricuspid annular plane systolic excursion (TAPSE), and left ventricular ejection fraction (LVEF). The TTA was measured in four chamber view as the maximal lateral diastolic distance at the level of an echocardiographically identifiable annulus. If a patient had a history of palpitations and/or the electrocardiogram showed arrhythmias, then electrophysiological studies were performed.

### Operative Procedure

**Fig. 1:**
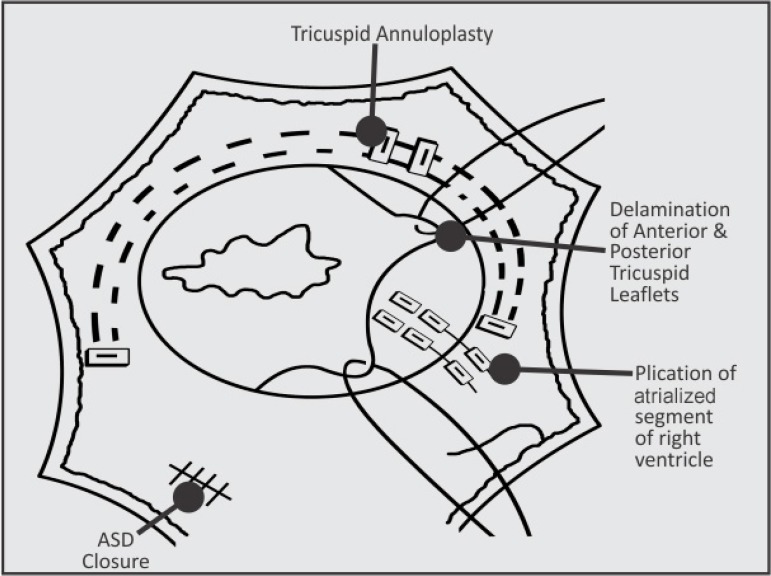
Diagrammatic presentation of repair - after delamination, atrialized RV is plicated in vertical fashion just closer to true tricuspid annulus. The true tricuspid annulus is circumferentially plicated using two 5-0 polypropylene suture to create a neoannulus. ASD=atrial septal defect

**Fig. 2:**
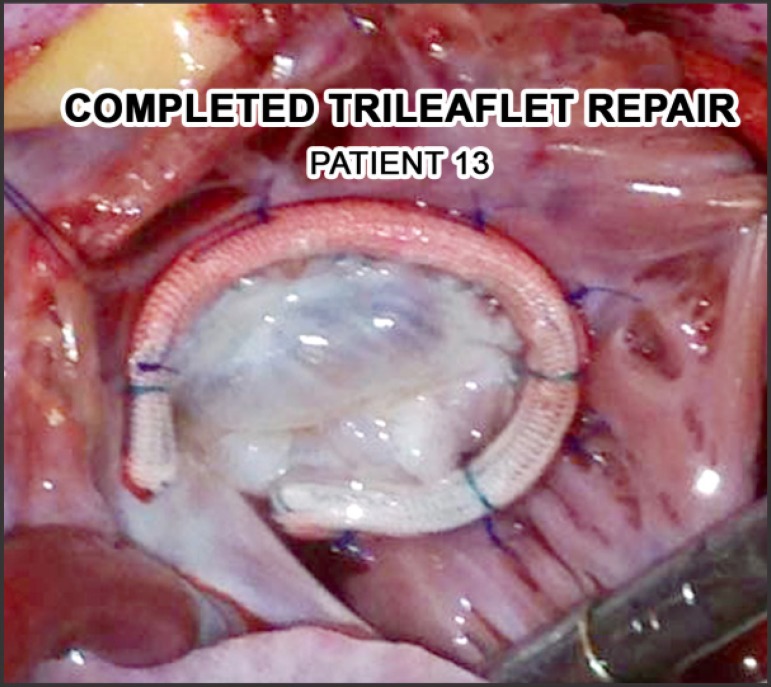
Intraoperative photograph of a completed Ebstein's repair showing neoannulus supported by a 3D ring and competent trileaflet repair.

Operation is performed via median sternotomy and cardiopulmonary bypass (CPB) is instituted with aortic, high superior vena cava and inferior vena cava cannulation. Intraoperative transoesophageal echocardiography is routinely used. Before initiating CPB, direct intraoperative pulmonary artery pressure is measured to confirm the feasibility of Glenn shunt (mean pulmonary artery pressure <15 mmHg). Moderate systemic hypothermia (32ºC) and antegrade cold blood cardioplegia are established. A standard oblique right atriotomy is performed with an incision from the right atrial appendage (RAA) towards the inferior vena cava, which is parallel to the right atrioventricular groove. The left heart is vented via patent foramen ovale or atrial septal defect. The tricuspid valve anatomy is examined using valve hooks and the atrialized RV is evaluated. Complete detachment and delamination of anterior tricuspid leaflet (ATL) and posterior tricuspid leaflet (PTL) are performed preserving some attachments to papillary muscles. The septal tricuspid leaflet (STL) is also detached and delaminated to reach the true annulus by 'somersaulting' of STL (releasing the lower margin of STL and flipping it over while intermittently retaining the upper margin's attachment to the septal wall). During delamination, as many chords as possible are retained after fenestration to be cut at a later stage if they prevent rotation of the leaflets around the annulus. Fenestrations present in ATL and PTL are closed using 6-0 polypropylene suture. After delamination, atrialized RV is plicated in vertical fashion just closer to the true annulus. A 75 ml, 26 Fr (Bard) Foley catheter is passed across the tricuspid valve and the balloon is slowly inflated to measure the right ventricular volume. Once measured, the balloon is deflated and the catheter is retrieved. The true annulus is circumferentially plicated using two 5-0 polypropylene sutures to create a 'neoannulus', thus reducing the area of tricuspid annulus to be covered by leaflet tissue (Figure 1). The detached leaflets are then rotated clockwise and attached to the neoannulus. Delamination of STL and somersault of STL will allow less tension on the posterior leaflet when rotated clockwise and prevents a taut PTL from creating a ledge in the TV´s inflow, which may give rise to gradients. The pre-reduction of TTA to create a smaller neoannulus allows us to always have adequate leaflet tissue to cover the tricuspid annulus, and we only rarely have to increase the septal leaflet with autologous glutaraldehyde-fixed pericardium. In any case, the trileaflet nature of the tricuspid valve is maintained as far as possible. Tricuspid valve competence is tested and the prolapsing segments are liberally supported by residual leaflet tissue or Gore-Tex neochordae arising from papillary muscle head (to allow elongation during growth in children). An appropriate size (usually 26 mm) of the 3D rigid ring (Medtronic) is seated with 5-0 polypropylene interrupted, non-pledgeted sutures, to support repair and to prevent late dilatation (Figure 2). The interatrial septum is closed. RAA is amputated and right atrial reduction is performed to stream inferior vena cava flow towards the tricuspid valve and avoid stasis of blood in right atrium, which can lead to thrombus formation. BCPS is performed in the routine fashion. CPB is discontinued. Inodilators (levosimendan/milrinone) and amiodarone infusion are used to stabilize the patient in the immediate postoperative period and to prevent and treat arrhythmias. All patients leave the operating room with a central venous pressure monitoring line through femoral vein and superior vena cava line to monitor Glenn pressure during the early postoperative period. Patients are routinely extubated early (<6 hours). Inodilators are tapered off one or two days after extubation. Postoperatively, patients are kept on amiodarone, beta-blocker, diuretics and angiotensin-converting-enzyme (ACE) inhibitors. Anticoagulation for 3 months and amiodarone for 6 weeks are continued postoperatively.

### Postoperative Management

Postoperative hospital mortality, intensive care unit (ICU) length of stay, postoperative hospital stay, reoperation for bleeding, renal failure, and any postoperative arrhythmias were observed. Postoperatively and at discharge echocardiographic TR was assessed by two independent operators and graded from 1 to 4. The highest grade was recorded as the TR degree. Follow-up was done at 1, 3 and 6 months postoperatively and 6 months later. At follow-up, New York Heart Association (NYHA) class of all patients was recorded. Echocardiographic evaluation was again performed by two independent operators for TR degree, TAPSE and BCPS patency.

### Statistical Analysis

The statistical calculations were performed using SPSS software v 20.0 (Chicago, IL, USA). Continuous and categorical data were expressed as mean ± SD and as proportions, respectively. The echocardiographic TTA 'Z' score preoperatively calculated was correlated with the intraoperative residual RV volume (after plication of the atrialized RV). The correlation between variables was calculated using Spearman's correlation coefficient. The cutoff value of *P*<0.05 was considered for statistical significance.

## RESULTS

From January 2012 to July 2016, 22 patients underwent surgical correction of Ebstein's anomaly. Median age was 12 years (ranging from 1.5 years to 27 years). Of the 22 patients, there were 8 (36.4%) females and 14 (63.6%) males. Cyanosis was present in 4 (18.2%) of the patients, however, almost all were desaturated with SPO_2_ around 90% (ranging from 50% to 94%). One (4.5%) of the patients on presentation had supraventricular tachycardia, which was evaluated by electrophysiological studies, but no aberrant pathway was found. The remaining patients at the time of presentation were in sinus rhythm. Four (18.2%) patients had associated ventricular septal defect, 18 patients had atrial septal defect/PFO and one (4.5%) patient had associated left superior vena cava draining into the coronary sinus. Echocardiographically, 4 (18.2%) patients were Carpentier type B, 17 (77.3%) patients were type C and one (4.5%) was type D. Mean TTA was 49.86±7.4 mm (ranging from 36 mm to 66 mm) with a mean 'Z' score of 3.72±0.57 (ranging from 2.32 to 5.28). Of the 22 operated patients, there were 13 (59.1%) with TTA 'Z' score of +2 to +4 and 9 (40.9%) with 'Z' score of ≥ 4. Ten (45.5%) patients had TR grade 3 and 12 (54.5%) had TR grade 4 (Table 1).

**Table 1:** Preoperative details of the study population.

Parameters		Value
Median age (years)		12 (range 1.5 to 27 years)
Sex (male)		14 (63.6%)
Symptoms	Cyanosis (n%)	4 (18.2%)
	Pedal edema (n%)	4 (18.2%)
	Hepatomegaly (n%)	4 (18.2%)
	Supraventricular tachycardia (n%)	1 (4.5%)
Associated cardiac defects	Ventricular septal defect (n%)	4 (18.2%)
	Atrial septal defect (n%)	18 (81.8%)
	Left superior vena cava (n%)	1 (4.5%)
Carpentier type	B (n%)	4 (18.2%)
	C (n%)	17 (77.3%)
	D (n%)	1 (4.5%)
NYHA class (mean ± SD)		2.59±0.7
Mean true tricuspid annulus (mean ± SD)		49.86±7.4 (range 36 to 66 mm)
True tricuspid annulus Z score (n%)	+2 to +4	13 (59.1%)
	≥4	9 (40.9%)
Tricuspid regurgitation (n%)	Grade 3	10 (45.5%)
	Grade 4	12 (54.5%)

NYHA=New York Heart Association

The mean functional NYHA class at presentation was 2.59±0.7. Two (9.09%) patients presented NYHA class IV with bilateral pedal edema and hepatomegaly and echocardiography showing TR grade 4. Of these two, one patient had previously undergone repair of the tricuspid valve with BCPS at another centre. He was re-repaired successfully by our technique. The other child, a 1.5-year-old male, had right heart failure, on high inotropic and ventilatory support preoperatively. This patient was Carpentier type D, presented tricuspid incompetence grade 4, with 'Z' score of TTA being +5.28. The patient could not be weaned off CPB after completion of the procedure and is the only mortality of our series.

**Fig. 3:**
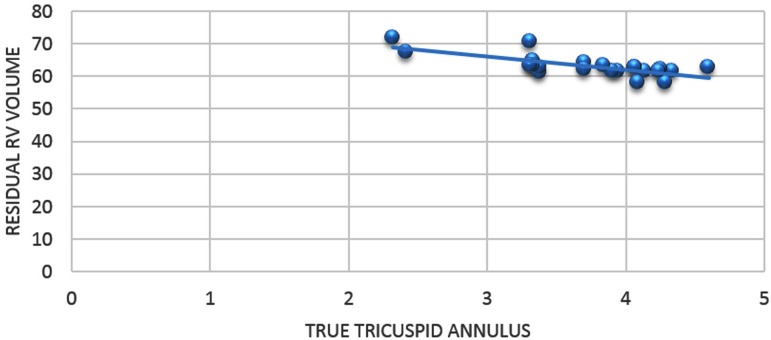
Correlation chart showing a negative correlation between TTA 'Z' score and percentage reduction in right ventricular volume following repair with a correlation coefficient of −0.83 (P value <0.01). RV=right ventricle; TTA=True tricuspid annulus

Intraoperatively, the right ventricular volume was measured as described above. This volume was then compared to the expected indexed right ventricular volume^[13]^. It shows that after plication of RV inferior aneurysmal wall, right ventricular volume is reduced to 71.96+3.8% of the expected volume. When correlated with the preoperative TTA 'Z' score, there was a significant negative correlation between TTA 'Z' score and the postplication indexed residual right ventricular volume, with a correlation coefficient of −0.83. It means that, as TTA 'Z' score increases, the indexed postplication residual RV volume reduces significantly (Figure 3).

Postoperatively, the mean duration of inotropes was 1.34±0.65 days and the mean postoperative ICU stay was 3.0±0.95 days. Mean postoperative hospital stay was 7.7±1.65 days. Postoperatively, one (4.5%) patient developed complete heart block, for which a permanent epicardial pacemaker implantation was performed later. Postoperative facial swelling and upper limb edema was noticed in only one (4.5%) case in the postoperative period, which was resolved on the 5^th^ postoperative day (Table 2).

**Table 2:** Postoperative details of the study population.

Inotrope duration (days)		1.34±0.65
Intensive care unit stay (days)		3.0±0.95
Hospital stay (days)		7.7±1.65
Complications (n%)	Bleeding	__
	Complete heart block	1 (4.5%)
	Facial edema	1 (4.5%)
	Arrhythmias	__
Follow-up period (months)		20.54±7.62

**Fig. 4:**
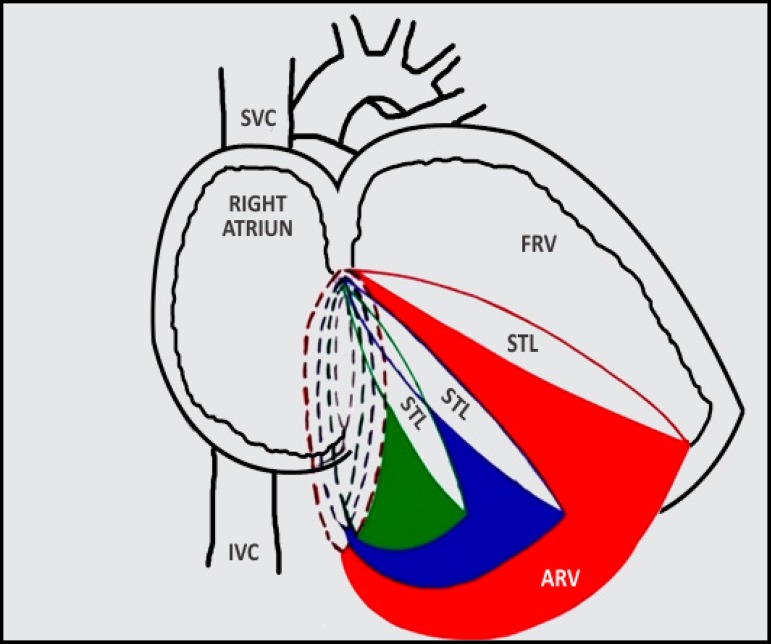
Schematic representation of severity of Ebstein's anomaly. As the displacement of septal tricuspid leaflet increases, there is a corresponding increase in true tricuspid annulus dilatation and a decrease in the amount of functional right ventricle. Broken lines indicate true tricuspid annulus. Coloured region indicates the thinned out atrialized right ventricle. Black - normal; green - mild dilatation; blue - moderate dilatation; and red - severe dilatation.

The follow-up was 100% complete with a mean follow-up period of 20.54±7.62 months (ranging from 10 to 36 months). There has been no late death to date. One (4.54%) patient needed reoperation for worsening NYHA class and increasing grade of tricuspid incompetence with dilatation of tricuspid annulus. This was a 1.5-year-old child who was operated according to our technique, except for non-placement of a tricuspid annuloplasty ring (the smallest annuloplasty ring of 26 mm was too large for him). Finally, the child underwent the Starnes procedure followed by extracardiac Fontan procedure. At the last follow-up, all patients were in NYHA class I or II. The functional class (NYHA) and TR grade had improved significantly (from 2.59±0.7 preoperatively to 1.34±0.52 and 3.40±0.65 preoperatively to 1.22±0.42 respectively, *P*<0.001). Mean TAPSE at follow-up was 16 mm compared to the preoperative mean TAPSE of 13.9 mm (*P*<0.0001) (Figure 4).

## DISCUSSION

In normal hearts, the septal and posterior leaflets are displaced downward in relation to the anterior mitral valve leaflet, but displacement is less than 8 mm/m^2^^[2]^. In Ebstein's anomaly, the displacement of the septal and posterior leaflets (> 8 mm/m^2^) ranges from very minimal to severe. This displacement effectively divides the ventricle into two regions, a part proximal to it that is functionally integrated with the right atrium and a part distal to it that is the effectively functional right ventricle.

Several studies have described natural history of Ebstein's anomaly^[5,14-16]^. Celermajer et al.^[5]^ found in their study that the actuarial survival rate was 67% at 1 year and 59% at 10 years. In childhood, adolescence and adult life, there was a continuous attrition related to hemodynamic deterioration and sudden and unexpected death. Even those who had an incidentally detected murmur had a small, but continuous, hazard for late death^[5]^. Hence, patients with Ebstein's anomaly require early surgery. The worst subset of patients presents earlier (*i.e.* infancy) and may more often Carpentier classification type D, compared to older children. Of all neonates with the diagnosis of Ebstein's anomaly, 20% to 40% did not survive 1 month, and <50% survive to 5 years^[5,17]^.

**Fig. 5:**
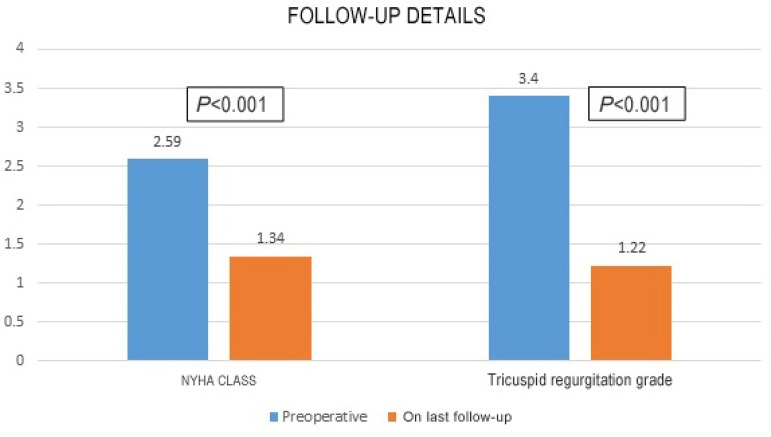
Comparison of pre- and postoperative data (follow-up).

Carpentier et al.^[4]^ and Celemajer et al.^[5]^ described the disease based on anatomical severity. However, although Carpentier classification^[4]^ describes anatomy well, it is not really prognostic, while the extended Glasgow Outcome Scale^[5]^ is prognostic, but very difficult to calculate. In Ebstein's anomaly, there is a disproportionate dilatation of atrialized RV with marked dilatation of the true tricuspid valve annulus (right atrioventricular junction)^[2]^. The more severe the displacement of the septal tricuspid valve leaflet, more is the atrialized right ventricle, which further dilates and leads to dilatation of the true tricuspid valve annulus (Figure 5). The true tricuspid annulus, although dilated and poorly defined, is not displaced and hence can be easily measured echocardiographically. We measured the intraoperative postplication indexed residual right ventricular volume and correlated it with the TTA 'Z' score. There is a significant negative correlation between the TTA 'Z' score and the postplication indexed residual right ventricular volume, with a correlation coefficient of −0.83. This means that, as TTA 'Z' score increases, the indexed postplication RV volume reduces significantly. Therefore, a high 'Z' score correlates with higher displacement, which results in a more aneurysmal dilatation of RV inferior wall (*i.e.* higher Carpentier type). Thus, we propose the measurement of this TTA by echocardiography as a surrogate marker for the severity of Ebstein's anomaly and suggest that its measurement be used as a guide to decide to add a BCPS. The TTA 'Z' score can be taken as a management tool for the patient with Ebstein's anomaly. TTA 'Z' scores ≥4 indicate an excessively dilated tricuspid annulus (and, by default, right ventricle). It is unlikely that this RV will be able to meet a complete cardiac output for long and will continue to dilate unless preload is reduced with a BCPS in addition to the tricuspid valve repair. For TTA 'Z' score of +2 to +4, we recommend the addition of BCPS, especially for patients with Carpentier type C and D for a long-term volume reduction and a potential RV reverse remodelling^[18]^. Tricuspid valve with a TTA 'Z' score ≤2 represents the 'forme fruste' of Ebstein's anomaly and does not need a BCPS if tricuspid valve repair is being done. The addition of BCPS is further supported by our observations: 1) after plication, the residual volume of RV is approximately 70% of the indexed RV volume expected; 2) The plication of the atrialized RV will lead to splinting effect on the inferior RV wall, which will not contribute to the RV ejection; 3) A dilated cardiomyopathic residual RV will not be functioning normally.

We recommend adding BCPS to tricuspid valve repair when:

- TTA 'Z' score of ≥4 with any Carpentier type;
- TTA 'Z' score is between +2 and +4 with Carpentier types C and D.

Malhotra et al.^[18]^ described cyanosis as the preoperative criterion to decide to add BCPS, otherwise, this decision was made intraoperatively after coming off bypass. However, we felt that an addition of BCPS should be an elective rather than a salvage decision. The moment the BCPS is salvaging the heart and repairing it, it means that the repair was reactive rather than proactive. In addition, it means that the heart already dilated too much to reverse remodel very well, even with preload reduction. Reverse remodelling of the heart is possible with a prophylactic BCPS when myocardial muscle fibres were not stretched beyond their maximum sarcomere length because at that stage myocardial fibres are no longer contractile and act like any other connective tissue fiber.

In our series, bidirectional Glenn was performed in 20 (90.9%) patients. The remaining two patients were Carpentier type B, with TTA 'Z' score of 2.2 and 2.1, requiring very minimal plication of the atrialized RV. Their postplication RV volume was 81% and 84% of the expected indexed RV volume. Therefore, BCPS was not performed. We observed that patients with type B generally have less displacement, leading to less atrialized RV and required less plication. Thus, BCPS should not be routinely performed in patients with type B class with a Z score of +2. We also observed that, after plication of the atrialized RV, indexed residual RV volume in our series was much lower than the expected indexed RV volume described in the literature^[13]^. This is another reason why we started BCPS when residual RV volume is less than 80% than expected.

In recent years, many authors have published successful management of Ebstein's anomaly using biventricular approaches^[19-21]^. In our series, the residual RV after plication of ARV was 70% of the expected indexed RV volume, which precludes a biventricular repair. Lange et al.^[21]^ have not added a BCPS to their repair, probably because of a different subset of patients in their series, as their postoperative RV end diastolic volume was close to normal. However, they left an interatrial communication of 5-6 mm in their patients, which would act as a trigger to decompress a failing RV. BCPS proposes to prevent precisely that, *i.e*., development of RV failure. Chauvaud et al.^[22]^ had 36% BCPS in their series, since their patient spectrum was much like ours.

Reduction in the functional RV load will have a significant effect on tricuspid valvular function^[23-25]^. A bidirectional cavopulmonary anastomosis will decrease the volume load on the already compromised right ventricular geometry in Ebstein's anomaly. Additionally, bidirectional cavopulmonary anastomosis will reduce transtricuspid valve flow and a more aggressive tricuspid annuloplasty may be performed, if required, in order to reduce TR to a minimum. Some series have, in fact, demonstrated that reduction of right ventricular preload alone may be sufficient to reduce TR to the point where no tricuspid valve intervention is required and maximum native valve structure can be preserved^[4,18,25]^.

One of the major concerns in performing a bidirectional cavopulmonary anastomosis is the effect of elevated pressure and pulsatility in the superior vena cava. However, in our study, facial swelling and upper limb edema was noticed in only one (4.54%) case in the postoperative period, which was also resolved on the 5^th^ postoperative day. Some authors also reported abnormal AV fistulas due to BCPS^[25]^ in their series, but we have not observed this complication.

Another concern of Ebstein's anomaly is the presence of a dilated atrialized right ventricle, which is a result of right ventricular cardiomyopathy^[26]^. In advanced cases, this dilated RV may cause bulging of ventricular septum leftward and cause compression of the left ventricular chamber^[2]^. Hence, plication of the atrialized RV during surgery will provide several advantages: 1) Reduction of non-functional portion of the right ventricle; 2) Better blood flow dynamics in right ventricle; 3) Reduction of left ventricular compression, thereby improving left ventricular function; 4) Elevating the papillary muscles, which facilitates the closure of the anterior leaflet against the septum in systole.

The one and a half ventricle repair for hypoplastic right heart is increasingly gaining a prominent role in the management of complex congenital heart diseases^[27,28]^. The Ebstein's malformation is one of the most suitable lesions to which this concept may be applied, especially because the pulmonary artery pressure in Ebstein's anomaly is never high. However, poor LVEF, mean PA pressure >20 mmHg, PVR of > 4 Wood units, LVEDP >12 mmHg preclude one and a half ventricle repair, as already mentioned by other authors^[18,25]^.

In Ebstein's anomaly, as the STL is displaced downwards, there is a discontinuity of central fibrous body and septal atrioventricular ring, which results in direct muscular connections between atria and ventricles. These may result in pre-excitation^[2,29]^. More than one accessory pathway is found in 6% to 36% of cases. Most of these accessory pathways are situated around the malformed tricuspid valve^[30-32]^. Antegrade and retrograde conduction through these fast conducting atrioventricular accessory pathways result in arrhythmias such as paroxysmal tachyarrhythmias, wide QRS tachycardia, ventricular tachycardia or flutter, atrial fibrillation or atrial flutter^[33,34]^. In our study, only one (4.5%) patient had supraventricular tachycardia. Although preoperative electrophysiologic studies did not reveal any accessory pathways in this patient, in the postoperative period, the patient recovered in sinus rhythm and did not have any episodes of SVT during follow-up. None of our patients demonstrated arrhythmias in the postoperative period. We presume that the plication of the true dilated tricuspid annulus and the dilated atrialized right ventricle, along with circumferential reattachment of disconnected ATL and PTL to the neoannulus, may interrupt some of these accessory pathways, which may help reduce the incidence of postoperative arrhythmias. In addition, RAA amputation and right atrial reduction would prevent arrhythmias originating from RAA due to right atrial dilatation.

In patients with Ebstein's anomaly, permanent postoperative pacing may be required in 3.7% of the cases^[35]^, most commonly for atrioventricular block and rarely for sinus node dysfunction. In our study, one (4.5%) patient needed a permanent postoperative pacemaker for complete heart block. The concern in performing BCPS is the lost access through superior vena cava for transvenous endocardial lead placement.

### Limitation

Our study limitation is the small number of patients, the lack of a control group and the short follow-up period. In addition, the long-term complications of bidirectional Glenn have to be keep in mind. Our use of the Foley catheter to measure RV volume may incur an error of approximately 10% in measurement. As other authors^[34,35]^ have produced good results with biventricular repair, our strategy has to stand the test of time. Magnetic resonance imaging evaluation of these patients during follow-up over a longer period could be more informative.

## CONCLUSION

Ebstein's anomaly is a right ventricular cardiomyopathy and truly a one and a half ventricle heart. A higher TTA 'Z' score correlates with a higher Carpentier class and lower functional RV volume. These patients always do better with complete trileaflet tricuspid repair and offloading of RV with BCPS. TTA 'Z' score ≥4, irrespective of the Carpentier type, should always require a BCPS, while Carpentier types C and D with TTA 'Z' score between +2 to +4 should also be considered for a BCPS (Table 3). Comprehensive repair is mandatory in specified subsets and provides good results. The short-term outcomes of our technique are promising.

**Table 3:** Indication for bidirectional cavopulmonary shunt.

	TTA 'Z' score <2	TTA 'Z' score 2-4	TTA 'Z' score >4
Carpentier type A/B	No BCPS	± BCPS	BCPS
Carpentier type C/D	No BCPS	BCPS	BCPS

TTA=true tricuspid annulus; BCPS=bidirectional cavopulmonary shunt

**Table:** 

Authors' roles & responsibilities	
AM	Substantial contributions to the conception or design of the work; or the acquisition, analysis, or interpretation of data for the work; drafting the work or revising it critically for important intellectual content; agreement to be accountable for all aspects of the work in ensuring that questions related to the accuracy or integrity of any part of the work are appropriately investigated and resolved; final approval of the version to be published
VA	Substantial contributions to the conception or design of the work; or the acquisition, analysis, or interpretation of data for the work; drafting the work or revising it critically for important intellectual content; agreement to be accountable for all aspects of the work in ensuring that questions related to the accuracy or integrity of any part of the work are appropriately investigated and resolved; final approval of the version to be published
KP	Substantial contributions to the conception or design of the work; or the acquisition, analysis, or interpretation of data for the work; drafting the work or revising it critically for important intellectual content; agreement to be accountable for all aspects of the work in ensuring that questions related to the accuracy or integrity of any part of the work are appropriately investigated and resolved; final approval of the version to be published
MS	Design of the work; or the acquisition, analysis; final approval of the version to be published
KS	Revising it critically for important intellectual content; final approval of the version to be published
PS	Revising it critically for important intellectual content; final approval of the version to be published
SS	Revising it critically for important intellectual content; final approval of the version to be published
NO	Revising it critically for important intellectual content; final approval of the version to be published
HP	Analysis, or interpretation of data for the work; final approval of the version to be published
